# Formula Labelling in the United Kingdom: Manufacturers' Compliance With the Code, UK Law and Guidance Notes

**DOI:** 10.1111/mcn.13794

**Published:** 2025-01-31

**Authors:** Maiko Kamata, Catherine Pereira‐Kotze, Marko Kerac, Victoria Sibson

**Affiliations:** ^1^ London School of Hygiene and Tropical Medicine London UK; ^2^ First Steps Nutrition Trust London UK

**Keywords:** CDH (Commercial Determinants of Health), commercial milk formula, enforcement, infant feeding, International Code of Marketing of Breastmilk Substitutes, labelling, legislation, marketing, monitoring, regulations

## Abstract

The International Code of Marketing of Breastmilk Substitutes (‘the Code’) was established to protect babies and young children from inappropriate marketing of formula milk, bottles and teats and avoid undue commercial influence on caregiver infant feeding practices, including undermining breastfeeding and safe and appropriate formula feeding. UK law encompasses some but not all of the Code. To address persisting concerns about the marketing of infant formula (IF) and follow‐on formula (FoF), we assessed labelling compliance in the UK against relevant provisions in the Code, UK law and Department of Health and Social Care (DHSC) Guidance Notes which interpret UK law. Data were collected during July and August 2022 by taking pictures of labels from company websites, in shops and online. We developed three labelling checklists to systematically assess compliance and to compare compliance scores between the regulatory frameworks, formula types and brands. We assessed 57 labels (*n* = 32 IF and *n* = 25 FoF) and found low overall compliance: 50% complied with UK law, 32% with Guidance Notes and 40% with the Code. None of the labels complied with provisions prohibiting idealising text and photographs, nutrition and health claims (where relevant) and cross‐promotion between formula types. In conclusion, UK IF and FoF labels violate many of the provisions of all three regulatory frameworks. This is evidence of inappropriate marketing. The UK law should be better enforced and strengthened in line with the Code to protect breastfeeding, support safer, appropriate formula feeding and lessen commercial influence on infant feeding practices.

## Summary

- Only 50% of 57 labels (*n* = 32 infant formula, *n* = 25 follow‐on formula) complied with UK laws, 32% with Department of Health and Social Care Guidance Notes, which interpret UK law, and 40% with the International Code of Marketing of Breastmilk Substitutes (‘the Code’).
- No labels complied with provisions prohibiting idealising text and photographs, nutrition and health claims and cross‐promotion between formula types.
- Poor compliance with labelling regulations indicates inappropriate marketing.
- The UK law should be better enforced and strengthened in line with ‘the Code’ to protect breastfeeding, support safer, appropriate formula feeding and lessen commercial influence on infant feeding practices.

## Graphical abstract

The International Code of Marketing of Breastmilk Substitutes informs UK legislation; both include formula labelling provisions to ensure safe and appropriate use and protect breastfeeding. We found compliance with the UK labelling regulations to be very poor. Proper enforcement and stronger regulatory controls are needed to protect infant health.

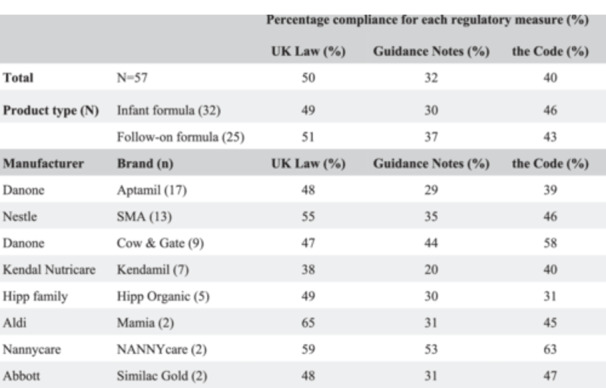

## 1 Introduction

Breastfeeding has multiple health benefits for both infants and mothers. Breastfed babies are protected from infections in early life and from obesity in later life; breastfeeding mothers have a lower risk of postpartum depression, breast and ovarian cancers and Type 2 diabetes (Victora et al. 2016). There are also major environmental and climate benefits. Formula milk generates gas emissions and plastic waste, whereas breastfeeding creates minimal pollution or waste (Long et al. 2021; Andresen et al. 2022).

The World Health Organization (WHO) recommends exclusive breastfeeding (EBF) to 6 months with the introduction of safe and adequate complementary foods at 6 months and continued breastfeeding up until 2 years and beyond (World Health Assembly [WHA] 2001). These recommendations are supported by the United Kingdom government (Scientific Advisory Committee on Nutrition [SACN] 2018, 2023). Yet, 2020/2021 data from England indicate that even by 6–8 weeks of age, EBF is only 36.5% (Office for Health Improvement and Disparities 2023) far below the global targets of 50% EBF to 6 months by 2025 and 70% by 2030 (World Health Organization [WHO] and United Nations Children's Fund [UNICEF] 2021).

One factor contributing to low breastfeeding prevalence is the active and forceful marketing of commercial milk formulas (CMF) (Unar‐Munguía et al. 2022; WHO and UNICEF 2022; Zhu et al. 2023). This has contributed to a doubling of formula sales over the past 20 years, to $55 billion annually worldwide (Baker et al. 2021). In a 2020–2021 multi‐country study conducted by the WHO and UNICEF, 51% of 8528 parents and pregnant women in the United Kingdom reported being exposed to ‘aggressive’ marketing by formula companies (M&C Saatchi World Services 2022; WHO and UNICEF 2022). Inappropriate marketing of CMF continues despite the adoption of the International Code of Marketing of Breastmilk Substitutes (the Code) by the World Health Assembly (WHA) over 45 years ago, and 19 subsequent WHA resolutions, which update the Code in response to contextual changes (United Nations Children's Fund [UNICEF] 2023). The Code aims to prevent commercial pressures from influencing infant feeding decisions—protecting breastfeeding as well as safer, appropriate formula feeding—by regulating the marketing, but not the availability, of breast milk substitutes (World Health Organization [WHO] 1981). However, globally, in May 2024, only 33 countries had national regulations that were substantially in line with the Code (World Health Organization (WHO), United Nations Children's Fund (‎UNICEF)‎, and International Baby Food Action Network (IBFAN) 2024), and company marketing practices in violation of the Code (including the advertisement of breast milk substitutes, lobbying of policymakers and sponsorship of healthcare professionals), remain widespread in almost all countries (Becker et al. 2022; WHO and UNICEF 2022). Code violations persist (especially cross‐promotion and through digital marketing) despite more countries implementing national legislation, due to poor compliance, monitoring and enforcement (Topothai et al. 2024), including inadequate penalties or sanctions in response to violations.

The CMF industry adversely influences public health since its commercial nature and main priority to increase profits through product sales, is inconsistent with public health priorities, where the protection of breastfeeding is a key to optimising maternal, infant and young child health (Baker et al. 2021). The way in which CMF, bottles and teats are marketed targets individuals and society and shape social norms, values and beliefs around infant feeding (WHO and UNICEF 2022; Baker et al. 2021). This undermines progress towards optimal breastfeeding and safer, appropriate formula feeding by distorting individual decision‐making and promoting the inappropriate use of CMF, including in circumstances where it may not be necessary or the best choice for babies or their mothers (Brown, Jones, and Evans 2020; Rollins et al. 2023; WHO and UNICEF 2022; WHO 2017).

Product labelling is a key marketing strategy since the information and direct/indirect messages conveyed are key to carers buying or not buying a particular CMF (Conway, Esser, et al. 2023; Conway, Ritchie, et al. 2023). Global evidence shows that CMF companies include aspirational texts and pictures and use health and nutrition claims on their product labels, including the implication that CMF may be equivalent or superior to breastfeeding (Cheung et al. 2023). Consequently, caregivers who rely on labels to understand product features, compare brands and make informed feeding decisions can be inadvertently influenced by these marketing tactics (Cheung et al. 2023; Conway, Esser, et al. 2023; WHO and UNICEF 2022). Cross‐promotion is another common marketing strategy that makes the brand the focus of the marketing and uses similar labelling, colours and logos across different products in the same line (WHO and UNICEF 2019; WHO 2017). This tactic builds brand loyalty and allows indirect promotion of products whose marketing is restricted, such as infant formula. Despite the Code explicitly prohibiting these marketing strategies, they are common in countries where national regulations are weaker than the Code (Cheung et al. 2023; Pereira et al. 2015).

In the European Union, Commission Delegated Regulation (EU) 2016/127 (CDR 2016/127) was adopted in 2016 and has been maintained in the United Kingdom since leaving the EU (Legislation.gov.uk. 2016). The CDR 2016/127 sets out specific rules on labelling, composition and marketing of infant formula and follow‐on formula, and is enforced by Regulation (EU) No. 609 of 2013 on Food for Specific Groups (FSG Regulation) (Legislation.gov.uk. 2020). The UK's Department for Health and Social Care (DHSC) provides Guidance Notes, which outline the DHSC's interpretation of national legislation to assist CMF companies, local governments and other interested parties so that they can fully implement the UK law (Department of Health and Social Care [DHSC] 2024). However, UK legislation only encompasses some of the provisions of the Code, illustrated by the UK scoring only 40 points out of 100 in the most recent international assessment of national implementation of the Code (WHO, UNICEF,‎ and IBFAN 2024). One major gap in UK legislation is that marketing of follow‐on formula for use from 6 to 12 months of age is permitted. This is despite guidance from the NHS that follow‐on formula is not necessary, and infants being fed with formula milk can continue to have infant formula (marketed for use from birth) until 1 year old (National Health Service [NHS] 2023). Even though UK legislation is relatively permissive compared to the Code, non‐compliance with the labelling restrictions that do exist has been documented, including through the use of health and nutrition claims, non‐permitted images and cross‐promotion (Conway, Ritchie, et al. 2023; WHO 2022). We undertook this study to further describe current compliance of infant formula and follow‐on formula labelling practices with the Code, as well as the UK law and the DHSC's interpretation of the law. By showing which provisions are poorly adhered to, we aimed to show where regulations and/or enforcement could be strengthened to improve protection against inappropriate marketing, to better protect infant and maternal health.

## 2 Methods

### 2.1 Study Design and Research Setting

This was a cross‐sectional study assessing labelling practices of all infant formula (marketed for use from birth) and follow‐on formula (marketed for use from 6 to 12 months) sold in the United Kingdom. Labelling practices were assessed for compliance with relevant provisions of the Code, UK law and Guidance Notes produced by the DHSC, which provide their interpretation of some provisions of the law.

### 2.2 Sample Selection

Eligible products were identified from the First Steps Nutrition Trust ‘Infant milk info’ website, which seeks to provide healthcare professionals supporting families with infant feeding with up‐to‐date and independent information on the formula products available on the UK market (First Steps Nutrition Trust n.d.). A small number of additional eligible products were identified from companies' official websites, based on inclusion and exclusion criteria described in Table 1. Many specific formula products are available in different formats (e.g., liquid, powder or tablets) or different unit sizes. During sample selection, we excluded duplicate products based on these variations (see Table 1).

**Table 1:** Inclusion and exclusion criteria.

Inclusion	
All infant formula and follow‐on formula sold in the United Kingdom	Where the different sizes of multipack products were available such as 4 × 200 and 6 × 200 mL bottles, one was chosen and assessed as they use the same labelling for different sizes.
	6 × 90 mL bottle pack products are only sold as a package; thus, both the cover package and the label on the bottle were assessed.
	For the sachets and tab^a^ formulations, the labelling on the outer carton only was assessed as the information on the inner package is duplicative/not additive or unique to the outer carton.
Exclusion	
Any formula milk marketed as foods for special medical purposes and growing‐up milk (suitable from 1 year onwards)	Hungrier baby formula, anti‐reflux formula, comfort formula, lactose‐free formula, hypoallergenic formula, soya formula and growing‐up milk (44).
	The items that were not available anymore or were out of stock at the time of sampling data were excluded.
	For multi‐bottle products, only cover packages such as paper covers and boxes were assessed. An individual bottle of the pack products was not assessed as they are also available for sale as a single product. They were included in the products assessment list as a single product.

^a^ A formula tab is a premeasured compact, single‐serving of formula milk powder.

While milk‐based drinks for children 1–3 years (marketed in the UK as ‘growing‐up’ and ‘toddler’ milks) are considered as breast milk substitutes by the WHO (2018), we excluded them from this research as there is currently no specific regulation governing this category of products in the United Kindom. CMF marketed as foods for special medical purposes (FSMPs) were also excluded since they are intended for use under medical supervision and by a very specific and small proportion of infants in the wider population.

### 2.3 Data Collection

Data were collected by one researcher during July and August 2022. Images of labels were obtained from publicly accessible company websites. When labels were not available online, photographs were taken in retail settings. Finally, if products were unavailable in shops, we purchased them online and assessed the label directly. Product label information, including text, images and other designs, was systematically extracted from labels and compiled into a Microsoft Excel spreadsheet to assess compliance (Yes/No) against each regulatory provision, ensuring data completeness and facilitating a systematic analysis.

### 2.4 Data Analysis

For assessing compliance with UK law, Commission Delegated Regulation (CDR) 2016/127 and Article 10 of the overarching FSG regulation, which are adopted in the EU, were used to develop the UK law checklist (see Supporting Information S1: Appendix 1.1) (Legislation.gov.uk. 2007, 2016). After identifying the provisions relating to labelling in UK law, the corresponding explanations on the Guidance Notes were extracted, and the Guidance Notes checklist was developed (see Supporting Information S1: Appendix 1.2) (Department of Health & Social Care 2022). We established the Code checklist by extracting provisions from the original Code in 1981 and 2022 WHO Europe policy brief on ‘Effective regulatory frameworks for ending inappropriate marketing of breast‐milk substitutes and foods for infants and young children in the WHO European Region’. This policy brief provides a ‘Model law for the WHO European Region’ which reflects the Code and all subsequent WHA resolutions (see Supporting Information S1: Appendix 1.3) (WHO 1981; World Health Organization Europe 2022). As a result, we identified 17 provisions in UK law, 10 in the Guidance Notes and 26 in the Code that were used to form the checklists against which each product label was assessed for compliance. For the UK law, six provisions are relevant just to infant formula, five just to follow‐on formula and six apply to both; for the Guidance notes, three provisions are relevant just to infant formula, one to follow‐on formula and six apply to both; whereas all 26 provisions in the Code apply to all products as no distinction is made between formula types (see Supporting Information S1: Appendices 1.1–1.3).

For provisions requiring the inclusion of a specific statement, compliance was categorised as Yes/No depending on whether the statement was included or not. For all other provisions, we established evaluation criteria to facilitate consistent and objective assessment of compliance (see Supporting Information S1: Appendices 1.1–1.3). After the first author assessed all products, five MSc Public Health students from the London School of Hygiene Tropical Medicine, recruited voluntarily through student networks, participated in structured online interviews to assess the consistency of assessment of compliance of product labels with regulatory frameworks. Six provisions from the UK law and two explanations from the Guidance Notes were selected for assessment as potentially subjective, as opposed to the other provisions requiring the inclusion or exclusion of specific content, which we viewed were objective. Disagreements between the first author and the students were resolved through discussion between the first and the second authors to finalise the assessment of compliance. This method acknowledges potential different interpretations between various assessors and mitigates the risk of subjectivity by triangulating the authors' perceptions with others.

Each label was given three compliance scores, one for each regulatory framework, expressed as percentage compliance with relevant provisions, as outlined above (for the UK law infant formula labels needed to Score 12 to be 100% compliant, and follow‐on formula needed to Score 11; for the Guidance Notes infant formula labels needed to Score 9 to be 100% compliant and follow‐on formula needed to Score 7 and for the Code both infant formula and follow‐on formula labels needed to Score 26 to be 100% compliant (see Supporting Information S1: Appendices 1.1–1.3).

We then examined how labelling compliance differed between regulatory frameworks, type of formula (infant formula compared to follow‐on formula) and the eight brands, by comparing mean percentage compliance scores for the labels assessed.

### 2.5 Ethics

Ethical approval was obtained from the London School of Hygiene and Tropical Medicine MSc Research Ethics Committee, LSHTM MSc Ethics Ref: 27092.

## 3 Results

### 3.1 Sample Characteristics

The labels of 57 products were assessed, of which 32 (56%) were infant formula and the rest (*n* = 25; 44%) were follow‐on formula. The brands (and manufacturing company) included were as follows: Aptamil (Danone, *n* = 17; 30%), SMA (Nestlé, *n *= 13; 23%), Cow & Gate (Danone, *n* = 9; 16%), Kendamil (Kendal Nutricare, *n* = 7; 12%), Hipp Organic (Hipp family, *n* = 5; 9%), Mamia (Aldi, *n* = 2; 4%), NANNYcare (Nannycare, *n* = 2; 4%) and Similac Gold (Abbott, *n* = 2; 4%) (Table 2).

**Table 2:** Percentage compliance with the labelling provisions of UK law, Guidance Notes and the Code by type of formula, manufacturer and brand.

		Percentage compliance for each regulatory measure (%)		
		UK law (%)	Guidance notes (%)	The Code (%)
Total	*N* = 57	50	32	40
Product type (*N*)	Infant formula (32)	49	30	46
	Follow‐on formula (25)	51	37	43

Manufacturer	Brand (*n*)	UK Law (%)	Guidance notes (%)	The Code (%)
Danone	Aptamil (17)	48	29	39
Nestle	SMA (13)	55	35	46
Danone	Cow & Gate (9)	47	44	58
Kendal Nutricare	Kendamil (7)	38	20	40
Hipp family	Hipp Organic (5)	49	30	31
Aldi	Mamia (2)	65	31	45
Nannycare	NANNYcare (2)	59	53	63
Abbott	Similac Gold (2)	48	31	47

### 3.2 Summary of Compliance With the Labelling Provisions of the UK Law, Guidance Notes and the Code

None of the labels complied with all of the labelling provisions of the UK law, Guidance Notes and/or the Code (Table 2). The highest mean compliance score among the three regulatory frameworks for the sample as a whole was for UK law, at 50%, while for the expanded provisions of the UK Guidance Notes, the score was 32% and for the Code, it was 40%. By formula type, compliance with UK law, Guidance Notes and the Code was 49%, 30% and 46% for infant formula and 51%, 37% and 43% for follow‐on formula, respectively. The labelling of follow‐on formula was more compliant with the UK law and Guidance Notes than infant formula labelling, while follow‐on formula labels were less compliant with the Code than infant formula labelling. By brand, overall, while NANNYcare labelling complied best with UK law, Guidance Notes and the Code at 55%, 53% and 61%, Kendal Nutricare labelling was the lowest at 38%, 20% and 40% compliance, respectively. Tables 3, 4, 5 show the compliance scores for each of the relevant provisions of the UK law, Guidance Notes and the Code.

**Table 3:** Compliance of product labels with the labelling requirements of UK law (Legislation.gov.uk. 2007, 2016).

Article/provision	No.	Checklist of labelling practices	Applicable to which formula milk type	Percentage compliant labels (*n*)
5(1)(2)	1	Names of infant formula and follow‐on formula. Are they appropriately labelled either ‘formula’ or ‘milk’ on the basis of their composition? − Entirely from cows' or goats' milk protein: described as ‘milk’ − Other than from cows' or goats' milk protein: described as ‘formula’	Both	100% (57)
6(2)	2	Does the product label of infant formula include the following		
(a)	2.1	A statement that it is suitable for infants from birth when they are not breastfed	IF	69% (22)
(b)	2.2	Instructions for appropriate preparation, storage and disposal of the product and a warning against the health hazards of inappropriate preparation and storage	IF	69% (22)
(c)	2.3	A statement that it should be used only on the advice of independent professionals. Independent professionals refer to people having qualifications in medicine, nutrition or pharmacy, or other professionals responsible for maternal and child care	IF	100% (32)
	2.4	A statement on 2.3 should be preceded by the words ‘important notice’ or their equivalent	IF	100% (32)
6(3)	3	Does the product label of the follow‐on formula include the following		
(a)	3.1	A statement that it is suitable only for infants over the age of 6 months	FoF	88% (22)
	3.2	A statement that it should form only part of a diversified diet	FoF	100% (25)
	3.3	A statement that it is not to be used as a substitute for breast milk during the first 6 months of life	FoF	96% (24)
	3.4	A statement that it should be used only on the advice of independent professionals	FoF	16% (4)
(b)	3.5	Instructions for appropriate preparation, storage and disposal of the product and a warning against the health hazards of inappropriate preparation and storage	FoF	64% (16)
6(5)	4	For the provision in Article 6, are all mandatory particulars easy to understand by the consumers?	Both	21% (12)
6(6)	5	Does the product label avoid using the terms ‘humanised’, ‘maternalised’, ‘adapted’ or terms similar to them?	Both	95% (54)
6(6)	6	Is the product labelling designed to avoid any risk of confusion between infant formula and follow‐on formula in terms of the text, images and colours used?	Both	0 (0)^a^
8	7	Does the product label avoid nutrition and health claims?	IF	100% (32)
9(3)	8	Does the information on DHA content appear with the required explanation (i.e., ‘as required by the legislation for all infant formula’)?	IF	3% (1)^b^
FSG 10(10)(1)	9	Is the product label designed so as not to discourage breastfeeding?	Both	0 (0)
FSG 10(10)(2)	10	Does the label avoid containing a photograph of the infant or any other photograph or text that would idealise the use of the products other than for explaining the preparation methods?	Both	0 (0)

Abbreviations: FoF, follow‐on formula; IF, infant formula.

a For *n* = 7, there was no counterpart to assess (no different type of formula in the same line in the same brand).

b For *n* = 2, labels did not refer to DHA at all.

**Table 4:** Compliance with the labelling requirements of Guidance Notes (Department of Health & Social Care 2022).

Article/provision	No.	Checklist of labelling practices	Applicable to which formula milk type	Percentage compliant labels (*n*)
6(2)(c)	1	Is ‘Important Notice’ clearly visible, prominent and understandable on the label?	IF	50% (16)
6(2)(b), (3)(b)	2	About a warning statement for the health hazards of inappropriate preparation and storage		
	2.1	Does the product label emphasise an increased risk, such as serious stomach upsets, diarrhoea and vomiting, constipation and dehydration and so on by inappropriate use?	Both	0 (0)
	2.2	Does the warning statement appear in a prominent place on the label, clearly visible and easily understood? DHSC suggests that it should have a contrasting font in respect of both size and colour.	Both	19% (11)
	2.3	Does the warning statement include wording such as ‘Failure to follow instructions may make your baby ill’?	Both	89% (51)
6(6)(b)	3	Are the labels of the infant formula and the follow‐on formula clearly differentiated from each other? All three of the text, images and colours used on the packaging must be different.	Both	0 (0)^a^
FSG 10(2)	4	Does the product label avoid containing pictures of an infant or any other pictures or text that would idealise the use of the product? DHSC provides examples of representations which may be considered to ‘idealise’ the products.	Both	0 (0)
	5	Are the specific terms ‘infant formula’ and ‘follow‐on formula’ clearly featured on the packaging as consumers may associate these terms with feeding infants from birth, whereas follow‐on formula should be used only from 6 months?	Both	100% (57)
	6	Does the product label contain or refer to breast milk or breastfeeding?	FoF	48% (12)
8	7	Does the product label avoid containing any nutrition and health claims on the labels? DHSC explains what nutrition and health claims are.	IF	0 (0)
9(3)	8	Does the information on DHA content appear with the required explanation (i.e., ‘as required by the legislation for all infant formula’)? DHSC suggests the text should be in close proximity to the area of the packaging, highlighting the presence of DHA.	IF	3% (1)^b^

Abbreviations: FoF, follow‐on formula; IF, infant formula.

a For *n* = 7, there was no counterpart to assess (no different type of formula in the same line in the same brand).

b For *n* = 2, labels did not refer to DHA at all.

**Table 5:** Compliance with the labelling requirements of the Code (WHO 1981; World Health Organization Europe 2022).

Article/provision	No.	Checklist of labelling practices	Percentage compliant labels (*n*)
9(1)	1	Is the product label designed to provide the necessary information about the appropriate use of the products, so as not to discourage breastfeeding?	7% (4)
9(2)	2	Does the product label explain the following in a clear, conspicuous and easily readable manner?	7% (4)^a^
	2.1	The words ‘Important Notice’ or their equivalent	53% (20)
	2.2	A statement of the superiority of breastfeeding	53% (30)
	2.3	A statement that the product should be used only on the advice of a health worker as to the need for its use and the proper method of use	32% (18)
	2.4	Instructions for appropriate preparation, and a warning against the health hazards of inappropriate preparation	16% (9)
	3	Does the product label avoid containing pictures of infants or pictures or text that idealises its use?	0 (0)
9(4)	4	Does the product label contain the following statements:	
	4.1	The ingredients used	100% (57)
	4.2	The composition/analysis of the product	100% (57)
	4.3	The storage conditions required	98% (56)
Resolution 5(1)	5	Does the product label avoid containing photographs, drawings or other graphic representations other than for illustrating methods of preparation?	0 (0)
Resolution 5(2)	6	Does the product label explain the following in a clear, conspicuous and easily readable manner?	0 (0)^b^
	6.1	Instructions for appropriate preparation and use in words and in easily understood graphics	40% (23)
	6.2	The age in numeric figures after which the product is recommended	0 (0)
	6.3	A warning about the health risks of improper use, preparation or storage and of introducing the product before the recommended age (for FoF)	19% (11)
	6.4	The required storage conditions both before and after opening, taking into account climatic conditions	34% (31)
	6.5	The name and national address of the manufacturer or distributor	93% (53)
Resolution 5(3)	7	Does the product label avoid containing any health or nutrition claims or state or imply that a relationship exists between the product or its components and health?	0 (0)
Resolution 6(1)	8	Does the product label contain the following	
	8.1	The words, ‘IMPORTANT NOTICE’ in capital letters and thereunder, ‘Breastfeeding is the normal and optimal way to feed infants and young children. Breast milk is important for the healthy growth and development of infants and young children. It protects against diarrhoea and other illnesses	0 (0)
	8.2	The word, ‘WARNING’ and thereunder, ‘Before deciding to supplement or replace breastfeeding with this product, seek the advice of a health professional. It is important for your baby's health that you follow all preparation instructions carefully. If you use a feeding bottle, your baby may refuse to feed from the breast. It is more hygienic to feed from a cup’	0 (0)
	8.3	Preparation instructions:	46% (26)^c^
		i. Powdered formula is not sterile and may be contaminated with pathogenic microorganisms during the manufacturing process or may become contaminated during preparation ii. It is necessary for the formula to be prepared one feed at a time using water first boiled and then cooled to not less than 70°C iii. Any unused milk must be discarded immediately after every feed	
	8.4	A feeding chart in the preparation instructions	81% (46)
	9	The product label avoids the use of the terms ‘maternalised’, ‘humanised’ or similar terms or any comparison with breast milk	95% (54)
	10	The product label avoids the use of text that may tend to discourage breastfeeding	0 (0)
	11	The specific source of the protein	84% (48)
	12	For FoF, does the product label state that it should not be used for infants less than 6 months old or used as the sole source of nutrition for infants?	88% (22)

a 23%, *n* = 13 partially compliant.

b 60% *n* = 34 partially compliant.

c *n* = 25 not applicable because not powder.

### 3.3 Cross‐Promotion

**Figure 1:**
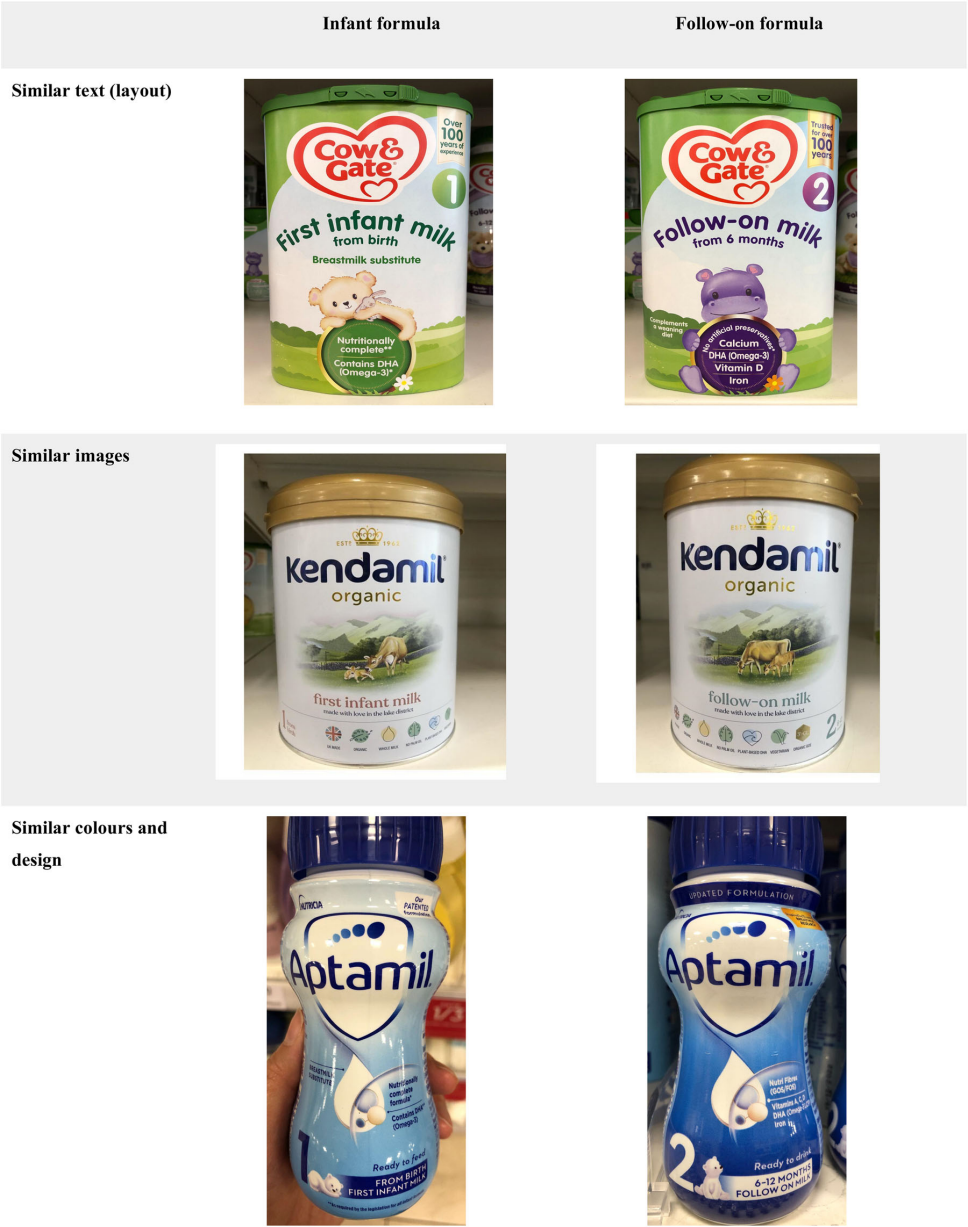
Examples of cross‐promotion between infant formula and follow‐on formula: noncompliance in labelling provisions with insufficient differentiation between infant formula and follow‐on formula.

Current UK law and DHSC Guidance Notes are clear regarding the requirement for differences in the text, images and colours of infant formula and follow‐on formula milk labels to prevent cross‐promotion. In this research, cross‐promotion was documented across all brand‐equivalent infant formula and follow‐on formula products as at least one out of the text, images or colours were similar. Examples of products that used similar labels are in Figure 1. It should be noted that while the UK law does not require numbering with a ‘stage’, all products were numbered 1 for infant formula and 2 for follow‐on formula, either with or without numerical age guidance, such as ‘from 0 months’ for infant formula. Additionally, some infant and follow‐on formula products were found to advertise the same line of the same brand of follow‐on formula or toddler or growing‐up milks, using photographs of the follow‐on formula, toddler or growing‐up milk on the label of the follow‐on formula (see Supporting Information S1: Appendix 2).

### 3.4 Nutrition and Health Claims

All infant formula labels used nutrition and/or health claims and were therefore in violation of current UK legislation. A frequent example was that 91% (*n* = 29) of infant formula labels included the phrase ‘nutritionally complete’. Others listed the presence of specific nutrients such as DHA, Omega‐3 and vitamin D, emphasising their role in being ‘nutritionally complete’ and in supporting the immune system. UK law does not prohibit nutrition and health claims on follow‐on formulas; however, these are not permitted by the Code. Some infant and follow‐on formula labels contained claims that emphasised the association between the product and health benefits, such as ‘support the normal function of the immune system’ (e.g., Aptamil Stage 2 Follow On Milk Powder and Tabs, Danone, Aptamil Organic Stage 2 Follow On Milk Powder, Danone) and ‘normal cognitive development’ (e.g., Aldi Mamia Follow on Milk Powder, Cow & Gate Stage 2 Follow‐on Milk Powder and Ready To Use, Danone) and some made claims focused on specific nutrients such as ‘Did you know that a 6‐month‐old baby's iron needs per kg/day are two times higher than a teenage girl?’ (SMA Advanced First Infant Milk Powder, Nestle).

### 3.5 Mandatory Information

All products complied with the provision of UK law that requires infant formula labels to include the phrase ‘important notice’ followed by mandatory particulars, including that the product is used under the advice of a healthcare professional. The Code also requires a follow‐on formula to have this phrase; however, only four of the packages (16.0%) complied. Other follow‐on formula labels stated: ‘The decision to start weaning, including the use of this product before 6 months, should only be made on the advice of a doctor….’. In this recommendation, the necessity of asking for professional advice before 6 months of age was clear, but it is unclear as to whether it is necessary to do so after 6 months of age.

The Code further requires IMPORTANT NOTICE to be prominently capitalised and for labels to include additional information on the benefits of breastfeeding. We identified only 42.1% (*n* = 24) of products using capital letters. Compliance was 100% for references to the superiority of breastfeeding, however, most brand labels used the same sentence: ‘breastfeeding is best’, and no specific health benefits were mentioned. All of this information was indicated on the side of the packaging and never on the front or back.

UK law requires a warning about health hazards associated with inappropriate preparation and storage of the product. All products explained that ‘Failure to [follow preparation instructions] may make your baby ill’; however, none described specific symptoms as required by the Code and Guidance Notes. Furthermore, compliance with the Code required the word ‘WARNING’ and the risk of impaired breastfeeding such as possible refusal to feed from the breast, which was not found on any labels.

All infant formula is required to include the statement ‘contains DHA’ on the label but only one infant formula contained information on the DHA content with the explanation required by UK law.

Finally, the Code mandates a feeding chart in the preparation instructions, although it does not specify the age range. Twenty‐six percent (*n* = 8) of infant formula provided a feeding chart only up to 6 months, even though they are suitable for use to 12 months of age. This gives the impression that the product is not suitable for use beyond 6 months, in a context in which the follow‐on formula is marketed for use from 6 to 12 months of age, and is subject to fewer legal marketing restrictions.

### 3.6 Label Content That Idealises Formula Milk and/or Discourages Breastfeeding

The UK law prohibits pictures and text that could idealise formula milk. Despite the explanation of what is considered to idealise the product given by the Guidance Notes, we identified this marketing tactic across all 57 products. Manufacturers used images of anthropomorphic characters and text suggesting the product as the ideal way for infant feeding by appealing to consumers' emotions. Table 6 provides descriptions of images and text that could be seen to idealise the products.

**Table 6:** Examples of idealising pictures and text that could discourage breastfeeding.

Example in guidance notes (Department of Health & Social Care 2022)	Examples of similar text and images found in product labels
Subjects related to babies and children, anthropomorphic characters, pictures and logos	Images Baby foot Baby animals: teddy bear, hippo, rabbit, goat and deer
Pictures or text that directly or indirectly refer to ‘the best’ or ‘the ideal method’ of infant feeding	Nutritionally complete Aptamil ‘ADVANCED’, SMA ‘Pro’ Personally guaranteed every product we make, GOLD AWARD Emphasis on the inclusion of specific nutrients and the health benefits of nutrients (nutrition and health claims)
Pictures or text implying that infant health, happiness or wellbeing, or the health, happiness and wellbeing of carers, is associated with infant formula or follow‐on formula. References to emotions of infants or carers	The beginning of your baby's life is a special and beautiful time For tips and non‐judgmental support, our specialist baby advisors and experienced mums are here to talk and encourage confident parenting at every stage. Serving millions of happy parents …has been crafted with the care and expertise that we believe every parent and baby deserves.
Text or pictures referring to ‘human milk’, ‘human milk oligosaccharides’, ‘breast milk’, ‘breastfeeding’, ‘moving on from breastfeeding’ or ‘closer to or inspired by breast milk’.	Inspired by 50 years of research into early life science Our expert team at Nutrition is dedicated to understanding the complex structure of breast milk and pursuing reflecting nature's wonderful nutrients in our own products. As identified within human breast milk

UK law also prohibits using the terms ‘humanised’, ‘maternalised’, ‘adapted’ or similar terms. While no products were identified using precisely these words, three products used ‘human’ as a synonym (e.g., ‘As identified within human breast milk’ and ‘Human milk oligosaccharides’).

### 3.7 Label Content Not Covered by Current Regulations

In assessing product labels, we also identified other non‐mandatory information not specified in the regulations but which could be considered as a type of marketing and against the Code. These include information on consumer helplines [on 97% (*n* = 55) of products\ and claims to the company's commitment to environmental issues (e.g., ‘we are taking… steps to reduce our impact and protect the planet…’) [on 49% (*n* = 28) of products].

## 4 Discussion

None of the 57 infant formula and follow‐on formula product labels we assessed fully complied with the labelling provisions of any of the three regulatory frameworks we applied to them, indicating that all CMF companies are in some way inappropriately marketing their products through their labelling practices. Compliance with labelling provisions in UK law was highest probably because they are legally binding, but it is important to note that these are also the least stringent among the three regulatory frameworks. Compliance with the Guidance Notes was lower than with UK law, most likely because the Guidance Notes contain stricter restrictions with detailed explanations of the UK law, plus they are non‐statutory (Department of Health & Social Care 2022). The Code provides the strictest restrictions, and UK law includes only some of the Code's provisions, so it is perhaps unsurprising that compliance of UK infant and follow‐on formula labels with the labelling provisions of the Code was lowest among the three regulatory frameworks (WHO 1981).

By formula type, follow‐on formula labelling complied better with UK law and Guidance Notes than infant formula labelling, while the opposite was the case when assessing compliance with the Code. This is because the UK law has stricter regulations for infant formula than for follow‐on formula. For example, advertising of follow‐on formula is permitted in the UK and the prohibition on nutrition and health claims only applies to infant formula (Legislation.gov.uk. 2016; Department of Health & Social Care 2022).

All products on the market in the UK displayed cross‐promotion between brand equivalent infant formula and follow‐on formula, all contained nutrition and health claims (legally on follow‐on formula, but against the Code) and all used texts and photographs idealising the product, in contravention of UK law (Legislation.gov.uk. 2016) and the Code.

### 4.1 Cross‐Promotion

In 2020, the UK law was updated to provide greater detail regarding the differentiation between infant formula and follow‐on formula required on labels (First Steps Nutrition Trust 2020a, 2020b). Despite this, none of the labels we assessed complied with this provision and all eligible products used either similar or identical colours, images or text on infant formula and follow‐on formula of the same brand range. This is consistent with an earlier study which evaluated labels of products available for sale in 2020 and reported that 72% of 18 follow‐on formula labels were nearly identical to infant formula labels, with more than four out of five of the features highlighted by the DHSC as needing to be different recorded as similar (Conway, Esser, et al. 2023).

All 57 product labels in our study were also seen to use the cross‐promotion tactic of numbering formula milk within a product line with a stage number, as opposed to age of use being presented with numerical figures as the WHO recommends (e.g., infant formula labelled 0–12 months). This marketing approach is designed to build loyalty to the entire range of products (WHO and UNICEF 2019), and during data collection, we observed that product ranges are sold next to one another in store, rather than being grouped by stage. Common branding and similar labelling across product lines (presented adjacent to each other in retail settings) confuse consumers, masking differences between products leading to inappropriate and unnecessary product choices, as well as undermining breastfeeding (WHO and UNICEF 2019; Berry, Jones, and Iverson 2010).

Follow‐on formula is often used after 6 months (McAndrew et al. 2012) when infant formula could continue to be used (NHS 2023) and then the ‘Stage 3’ growing‐up or toddler milk is used from 12 months (SACN 2023), when ordinary cows' milk could be provided (NHS 2023). National survey data (McAndrew et al. 2012) and other published studies also show the potentially harmful use of these products for children who are below the recommended age. A 2014 study in Italy showed that two‐thirds of mothers did not understand the meaning of the number ‘2’ on the follow‐on formula packages, with 28% interpreting that it could be used before 6 months of age (Cattaneo et al. 2015). Another study from South Africa reported that the ‘4’ was interpreted as the use of the product for 4 months when the product was recommended for 36 months. Follow‐on formula contains more iron, which may cause negative consequences (such as reduced uptake of other trace metals and the oxidation of lipids) with no developmental or growth advantages (First Steps Nutrition Trust 2020a; NHS 2019).

Our observation that formula companies use the labels of infant formula to advertise their ‘next stage’ formula (follow‐on formula) shows how the industry exploits the legal loophole which permits follow‐on formula marketing. In addition, since companies are legally allowed to run price promotions on a follow‐on formula, this is a strategy which can encourage more long‐term product purchasing to increase sales (First Steps Nutrition Trust 2023). This is a significant shortcoming in UK law, creating an important discrepancy with WHO recommendations. It also presents significant national policy incongruence given the NHS clearly states that the follow‐on formula is unnecessary, yet UK legislation allows for it to be advertised.

### 4.2 Nutrition and Health Claims

The use of nutrition and health claims on all infant formula and follow‐on formula labels is consistent with an earlier UK study (Conway, Ritchie, et al. 2023). Nutrition and health claims confuse consumers by giving the impression that formula is equal or superior to breastfeeding (Brown, Jones, and Evans 2020; Hughes, Landa, and Sharfstein 2017). Such claims often include selectively biased information with limited scientific evidence for marketing purposes rather than providing accurate information (Crawley and Westland 2016), undermining breastfeeding.

### 4.3 Mandatory Information

In line with UK law, the title ‘Important Notice’ was used in all products where it stated the superiority of breastfeeding, but a minority used capital letters or placed in a prominent position as required in the Code. All 57 products included the slogan ‘breastfeeding is best’ on their labels, but no products explained any further information about specific health benefits as required by the Code. We observed a similar trend in warnings of health hazards. No products described what possible symptoms a baby may experience in case of improper reconstitution, or of the potential negative effects of using it incorrectly. Infections caused by Cronobacter sakazakii are rare but can cause severe illness and death in infants (Food and Agricultural Organisation [FAO]/World Health Organization [WHO] 2006). Over 90% of Cronobacter infections in neonates are linked to powdered infant formula (PIF). Contamination of PIF can occur at the manufacture, reconstitution or storage of reconstituted products. Therefore, is it essential that PIF producers adhere to regulatory standards and that consumers adhere to FAO/WHO guidelines for PIF reconstitution and storage (Kalyantanda, Shumyak, and Archibald 2015). This could be facilitated by ensuring sufficient mandatory preparation, storage and safety information on product labels. It has been suggested that formula companies avoid emphasising the benefits of breastfeeding, or stating the risks associated with their products because consumers would have a negative impression of the products, with detrimental effects on sales (Hastings et al. 2020).

### 4.4 Images and Text Idealising Products

Despite UK law prohibiting the use of text and images that would idealise infant formula products to promote sales, we found these on all product labels. Other research in the UK found similar results, with idealising images on 67% of infant formulas and 78% of follow‐on formulas (Conway, Esser, et al. 2023). Using unnecessary emotional text and images that enhance a company's image and its products is a manipulative marketing tactic (Hastings et al. 2020).

### 4.5 Label Content Not Covered by Current Regulations

Our study revealed the company's exploitation of legal loopholes on infant and follow‐on formula labelling to further product marketing, including appealing to consumer concerns about the environment and advertising consultation services to parents/carers. ‘Greenwashing’ is a marketing strategy which encourages consumers to buy from eco‐friendly brands through disseminating false or misleading information about companies' environmental commitments (Competition and Markets Authority 2021). The provision of company care lines and similar approaches to engage parents/carers enables companies to obtain customers' personal information and use it for marketing purposes to increase long‐term sales (Hastings et al. 2020).

### 4.6 Strength and Limitations

This is the first study of UK infant and follow‐on formula labels which evaluates compliance with both UK legislation and the Guidance Notes designed to interpret the law, as well as the Code. The data was used to assess compliance with labelling provisions of each policy framework, and we present percentage compliance scores and descriptive data on what labelling approaches formula companies use to breach regulations. As a result, this study not only identifies areas of good and poor compliance but also what further clarifications and strengthening of regulations are required.

The main limitation of our study is that compliance with some provisions of the regulations is not perfectly objective; for example, what is perceived as idealising text. Furthermore, some may be better assessed by potential consumers; for example, provisions that require ‘easy‐to‐understand’ instructions. All products could be evaluated equally rather than subjectively if the government were to establish uniform criteria for classifying compliance. Additionally, FSMPs were excluded with justification, but some formula products marketed as FSMP are widely available to the public and also poorly comply with relevant labelling regulations, including making misleading claims on their labels, for example, comfort milk claim to ease colic and constipation (Baby Feeding Law Group United Kingdom [BFLG‐UK] 2022). Finally, while this is a cross‐sectional study, and label design is constantly changing and evolving with new product development we feel it is unlikely that the overall pattern of compliance will change substantially without a change in regulations and enforcement.

The focus of this research was labelling, but marketing is increasingly immersed in a rich digital marketing environment which was not examined and could be the focus of future research.

### 4.7 Recommendations

The UK's departure from the EU provides an opportunity to strengthen UK legislation to align with global guidance, that is, the Code, especially where significant national policy incongruence exists between Government recommendations on safer, appropriate formula feeding and the availability of products and their labelling; for example, the NHS clearly states that follow‐on formula is unnecessary, yet UK legislation allows for it to be marketed. This misalignment needs to be corrected to safeguard infant health; the DHSC should revise UK legislation as a matter of urgency to prohibit both follow‐on formula and growing‐up/toddler milk marketing. This is especially important because the WHO defines breast milk substitutes to include all CMF marketed up to the age of 36 months (WHO 2018), and in line with the Code, many countries restrict the marketing of both follow‐on formula and growing‐up milks (WHO, UNICEF, and IBFAN 2024). Furthermore, the UK Scientific Advisory Committee on Nutrition recently recommended the following: ‘Formula milks (including infant formula, follow‐on formula, ‘growing‐up’ or other ‘toddler’ milks) are not required by children aged 1 to 5 years’ (SACN 2023).

We should also broaden our view to encompass all marketing techniques, including unnecessary content on product labels such as promotion of customer care lines and greenwashing to enhance companies' images.

As well as closing legal loopholes and strengthening UK law in line with the Code, we recommend the Government implements independent monitoring and consistently enforces company compliance with legislation. Our results show that even the current limited legislation is not properly followed; for example, nutrition and health claims were found on infant formula labels and provisions on mandatory labelling information on health risks associated with inappropriate use of the products were not always present. It seems particularly important to address the latter given recent reports from the US documenting infant hospitalisations and deaths related to bacterial contamination of infant formula (Centres for Disease Control and Prevention 2022; U.S. Food & Drug Administration 2022) as well as increasing occurrences of climate hazards (Inter‐governmental Panel on Climate Change [IPCC] 2023), that may result in power outages or water shortages challenging safe formula preparation and use.

Further research is required into how the various marketing and advertising practices (including those used on product labels) specifically influence individual choices around infant feeding practices and product purchasing patterns. Other research has, for example, found targeted advertising aimed at healthcare professionals, (Hickman et al. 2021), plus authors of the 2023 Lancet Breastfeeding Series concluded that ‘Actions are needed across different areas of society to better support mothers to breastfeed for as long as they want, alongside efforts to tackle exploitative formula milk marketing once and for all’ (WHO 2023).

## 5 Conclusion

Infant formula and follow‐on formula labels are poorly compliant with the UK law, Guidance Notes and the Code indicating that CMF manufacturers are using product labels as a marketing tool to increase sales. Such tactics may be detrimental to mothers' and infants' health by distorting parents' and carers' decision‐making on what and how they feed their babies. Strengthening of the UK law, as well as active independent monitoring and enforcement of the regulations, are urgently required to ensure alignment with Government guidance on infant and young child feeding, as well as with WHO recommendations. A stronger legislative environment would ultimately better protect infant and young child health.

## Author Contributions

Maiko Kamata contributed to methodology, investigation, data curation, formal analysis, writing original draft and writing review and editing. Catherine Pereira‐Kotze contributed to supervision, formal analysis, writing review and editing. Marko Kerac contributed to supervision, methodology, formal analysis and writing review and editing. Victoria Sibson contributed to conceptualisation, supervision, methodology, investigation, data curation, formal analysis and writing review and editing.

## Conflicts of Interest

The authors declare no conflicts of interest.

## Supporting information

Supporting information.

## Data Availability

Data that support the findings of this study are available from the corresponding author upon reasonable request.
