# Supplementary material for: Formula Labelling in the United Kingdom: Manufacturers' Compliance With the Code, UK Law and Guidance Notes
Source: Matern Child Nutr. 2025 Jan 31;21(2):e13794. doi: 10.1111/mcn.13794 (PMC11956058; doi:10.1111/mcn.13794)
Supplement: Supplementary file 1 — Supporting information. [file MCN-21-e13794-s001.docx]

**APPENDICES**

Appendix 1.1 UK law evaluation criteria of labelling practices (CDR 2016/127, 2016; The Infant Formula and Follow-on Formula (England) Regulations 2007, 2007)

| **UK Law** | | | |
| --- | --- | --- | --- |
| **No** | **Checklist of labelling practices** | **Criteria for choosing answers:** | **Compliance** |
| 1 | Names of infant formula and follow-on formula. | Where label appropriately describes either 'formula' or 'milk' on the basis of their composition  - entirely from cows’ or goats’ milk protein: described as ‘milk’  - other than from cows’ or goats’ milk protein: described as ‘formula’ | Yes |
|  |  | Others | No |
| 2.1  2.2  2.3  2.4 | Does the product label of infant formula include the following: | Label provides at least one message for each of the following:  2.1 A statement that it is suitable for infants from birth when they are not breastfed  2.2 Instructions for appropriate preparation, storage and disposal of the product and a warning against the health hazards of inappropriate preparation and storage  2.3 A statement that it should be used only on the advice of independent professionals. Independent professionals refer to people having qualifications in medicine, nutrition or pharmacy, or other professionals responsible for maternal and child care  2.4 A statement on 2.3 should be preceded by the words ‘important notice’ or their equivalent | Yes |
|  |  | Label dose not mention above respectively | No |
| 3.1  3.2  3.3  3.4 | Does the product label of follow-on formula include the following:  3.1 A statement that it is suitable only for infants over the age of six months  3.2 A statement that it should form only part of a diversified diet  3.3 A statement that it is not to be used as a substitute for breast milk during the first six months of life  3.4 A statement that it should be used only on the advice of independent professionals | Lavel provides a statement of 3.1-3.4 respectively | Yes |
|  |  | Label dose not provide a statement of 3.1-3.4 respectively | No |
| 2.2  3.5 | Instructions for appropriate preparation, storage and disposal of the product and a warning against the health hazards | Label provides at least one message for all four information of preparation, storage, disposal and health hazards. | Yes |
|  |  | - Where one or more of the four messages are missing  - Where the information listed on the product is lacking for proper preparation, storage and disposal (e.g. there are no instructions on how to heat up despite the microwave not being available) | No |
| 3.4 | Does the product label of follow-on formula include the following:  A statement that it should be used only on the advice of independent professionals | Label clearly states the need for professionals advice on the use of products | Yes |
|  |  | Where the need for professional advice on the use of the product is unclear (e.g. 'the decision to start weaning including the use of this product before 6 months should be made only on the advice of a doctor' - Unclear need for professional advice on the use of the product after 6 months) | No |
| 4 | For the provision on Article 6, are all mandatory particulars easy to understand by the consumers? | Label classified all Yes for 2.1-2.4 for IF or 3.1-3.5 for FoF and no elements classified as No or Partial | Yes |
|  |  | - Where more than one of the 2.1-2.4 for IF or 3.1-3.5 for FoF are classified as No.  - Where there are uncertain information and not easy to undestand on 2.1-2.4 for IF or 3.1-3.5 for FoF such as no specific time to discard but it explains ‘it should discard immediately’, without other elements classifies as No | No |
| 5 | Does the product label avoid using the terms ‘humanised’, ‘maternalised’, ‘adapted’, or terms similar to them? | Where label does not use ‘humanised’, ‘maternalised’, ‘adapted’ and their synonyms | Yes |
|  |  | Where label exactly uses the words ‘humanised’, ‘maternalised’, ‘adapted’ and their synonyms such as ‘human’ | No |
| 6 | Is the product labelling designed to avoid any risk of confusion between infant formula and follow-on formula in terms of the text, images and colours used? | Where text, image and colours all different | Yes |
|  |  | Where any one of the text, image or colour is the same | No |
|  |  | Where there is no counterpart (no different type of formula in the same line in the same brand) | NA |
| 7 | Does the product label avoid nutrition and health claims? | Where label does not contain nutrition and health claims | Yes |
|  |  | Where label contains nutrition and health claims | No |
| 8 | Does the information on DHA content appear with the following statements? | Where the required statement (contains Docosahexaenoic acid (as required by the legislation for all infant formulae)' or 'contains DHA (as required by the legislation for all infant formulas) is listed in close to the DHA content | Yes |
|  |  | - Where the required statement is not listed in close to the DHA content  - Where label does not have the required statement | No |
| 9 | Is the product label designed so as not to discourage breastfeeding? | Other than those classified as No | Yes |
|  |  | - Where label contains nutrition and health claims  - Where label contains a photograph (other than for exlaining the preparation methods) or text that would idealise the use of the products (see No. 10) | No |
| 10 | Does the label avoid containing a photograph of the infant or any other photograph or text that would idealise the use of the products other than for explaining the preparation methods? | Other than those classified as No | Yes |
|  |  | - Where label contains nutrition and health claims  - Where label contains a picture  - Where label contains emotional text to ensure the quality of the product, to support consumers or to appeal the product or company  - Where label refers to breastfeeding or breastmilk | No |

Appendix 1.2 DHSC Guidance Notes evaluation criteria of labelling practices (DHSC, 2022).

| **Guidance Notes** | | | |
| --- | --- | --- | --- |
| **No** | **Checklist of labelling practices** | **Criteria for choosing answers:** | **Compliance** |
| 1 | Is ‘Important Notice’ clearly visible, understandable and prominent on the infant formula label? | Where all three items were classified as Yes  - Clearly visible: whether it uses the words "Important Notice" or their equivalent  - Understandable: whether the text of Important Notice is easy to understand in English  - Prominence: whether it is described in a different colour, size, font or capital letters | Yes |
|  |  | Any one of the three items is classified as No | No |
| 2.1 | Does the product label emphasise an increased risk such as serious stomach upsets, diarrhoea and vomiting, constipation and dehydration, and so on by inappropriate use? | Where label explains a warning statement about the health hazards including specific symptoms such as serious stomach upsets, diarrhoea and vomiting, constipation and dehydration, and so on | Yes |
|  |  | - Where label does not explain any specific symptoms caused by incorrect preparation  - Where label does not provide any information about a warning statement at all | No |
| 2.2 | Does the warning statement appear in a prominent place on the label, clearly visible and easily understood? DHSC suggests that it should have a contrasting font in respect of both size and colour. | Where both size and colour differ | Yes |
|  |  | Where either size or colour differs or both are the same | No |
| 2.3 | Does the warning statement include wording such as ‘Failure to follow instructions may make your baby ill’? | Where label includes a statement of 'Failure to follow instructions may make your baby ill’ or similar | Yes |
|  |  | Where label does not include a statement of 'Failure to follow instructions may make your baby ill’ or similar | No |
| 3 | Are the labels of the infant formula and the follow-on formula clearly differentiated from each other? All three of the text, images and colours used on the packaging must be different. | Where text, images and colours all differ. This also includes differences in the layout of text and images, and differences in the font of text. | Yes |
|  |  | Where label uses partially different text, images or colours | No |
|  |  | Where there is no counterpart to compare | NA |
| 4 | Does the product label avoid containing pictures of an infant or any other pictures or text that would idealise the use of the product? DHSC provides examples of representations which may be considered to ‘idealise’ the products. | Where label does not include an example or similar text and photograph of an idealised product provided in the Guidance Notes (See TABLE 5) | Yes |
|  |  | Where label includes an example or similar text and photograph of an idealised product provided in the Guidance Notes | No |
| 5 | Are the specific terms ‘infant formula’ and ‘follow-on formula’ clearly featured on the packaging? | Where label describe as infant formula’ or ‘follow-on formula’ with different colour from the background, size or font | Yes |
|  |  | Others | No |
| 6 | Does the product label contain or refer to breastmilk or breastfeeding? | Where label does not refer to breastmilk or breastfeeding | Yes |
|  |  | Where label refers to breastmilk or breastfeeding | No |
| 7 | Does the product label avoid containing any nutrition and health claims on the labels? DHSC explains what nutrition and health claims are. | Where label does not contain nutrition and health claims |  |
|  |  | Where label does not contain nutrition and health claims |  |
| 8 | Does the information on DHA content appear with the required explanation (i.e. ‘as required by the legislation for all infant formula’)? DHSC suggests the text should be in close proximity to the area of the packaging, highlighting the presence of DHA. | Where the required statement is listed in close to the DHA content | Yes |
|  |  | - Where the required description is explained elsewhere than in the DHA description  - Where label does not have the required statement | No |
|  |  | Where it does not refer to DHA at all | NA |

# Appendix 1.3 The Code evaluation criteria of labelling practices (WHO, 1981; WHO EU, 2022)

| **The Code** | | | | |
| --- | --- | --- | --- | --- |
| **No** | **Checklist of labelling practices** | **Criteria for choosing answers:** | | **Compliance** |
| 1 | Is the product label designed to provide the necessary information about the appropriate use of the products, so as not to discourage breastfeeding? | Where label is classified as Yes on 2.5 | | Yes |
|  |  | Where label is classified as Partial or No on 2.5 | | No |
| 2 | Does the product label explain the following in clear, conspicuous, and easy to read and understand in English: | Where label is classified as Yes for all 2.1-2.4 | | Yes |
|  |  | Where label is classified as partially Yes for 2.1-2.4 | | Partial |
|  |  | Where label is classified as No for all 2.1-2.4 | | No |
| 2.1 | The words "Important Notice" or their equivalent | - Clear: whether it clearly states ‘Important Notice’ or their equivalent  - Conspicuous: whether it is described in a different colour, size, font or capital letters  - Easily to read: whether it is easy to understand without any uncertainties | Where all three items were classified as Yes | Yes |
|  |  |  | Where some of the three items were Yes, or none of them was classified as Yes | No |
| 2.2 | A statement of the superiority of breastfeeding | - Clear and Conspicuous: whether it is clear and conspicuous is classified the same as 2.1 as the statement is described under the Important Notice.  - Easily to read: whether it is easy to understand without any uncertainties | Where all three items were classified as Yes | Yes |
|  |  |  | Where some of the three items were Yes, or none of them was classified as Yes | No |
|  |  | When label does not refer to it at all | | No |
| 2.3 | A statement that the product should be used only on the advice of a health worker as to the need for its use and the proper method of use | - Clear and Conspicuous: whether it is clear and conspicuous is classified the same as 2.1 as the statement is described under the Important Notice.  - Easily to read: whether it is easy to understand without any uncertainties | Where all three items were classified as Yes | Yes |
|  |  |  | Where some of the three items were Yes, or none of them was classified as Yes | No |
|  |  | When label does not refer to it at all | | No |
| 2.4 | Instructions for appropriate preparation, and a warning against the health hazards of inappropriate preparation | - Clear: whether it has a title such as ‘Preparing feeding.’  - Conspicuous: i) (whether it is explained in graphics for preparation OR used in a different colour, size or font for the title of preparation) AND ii) (used in a different colour, size or font for warning statement)  - Easy to read: whether it is easy to understand (e.g. if it does not explain how to warm or specific timing for discard of the product, it was classified as ‘No’) | Where all three items were classified as Yes | Yes |
|  |  |  | Where some of the three items were Yes, or none of them was classified as Yes | No |
|  |  | When label does not refer to health hazards | | No |
| 3 | Does the product label avoid containing pictures of infants or pictures or text that idealise the use of the product? | Where label does not contain designs that would idealise the use of formula (See the next row) | | Yes |
|  |  | - Where label contains nutrition and health claims  - Where label contains a picture  - Where label contains emotional text to ensure the quality of the product, to support consumers or to appeal the product or company  - Where label refers to breastfeeding or breastmilk | | No |
| 4 | Does the product label contain the following statements:  4.1 The ingredients used  4.2 The composition/analysis of the product  4.3 The storage conditions required | Where label provides a statement of 4.1-4.3 respectively | | Yes |
|  |  | Where label does not provide a statement of 4.1-4.3 respectively | | No |
| 5 | Does the product label avoid containing photographs, drawings or other graphic representation other than for illustrating methods of preparation? | Where label does not contain photographs, drawings or other graphic representation other than for illustrating methods of preparation | | Yes |
|  |  | Where label contains photographs, drawings or other graphic representation other than for illustrating methods of preparation | | No |
| 6 | Does the product label explain the following in a clear, conspicuous and easily readable manner, in English: | Where label is classified as Yes for all 6.1-6.5 | | Yes |
|  |  | Where label is classified as partially Yes for 6.1-6.5 | | Partial |
|  |  | Where label is classified as No for all 6.1-6.5 | | No |
| 6.1 | Instructions for appropriate preparation and use in words and in easily understood graphics; | Clear: whether it is explained in words and graphics without unambiguous  Conspicuous: whether it has a title such as ‘Preparing feeding’  Easily readable: whether it is easy to find and understand (e.g. when it does not explain how to warm, specific timing for discard, it was classified as ‘No’) | Where all three items were classified as Yes | Yes |
|  |  |  | Where some of the three items were Yes, or none of them was classified as Yes | No |
| 6.2 | The age in numeric figures after which the product is recommended | Where the age after which the product is recommended describes in numeric figures, and 'from birth' for infant formula and 'from 6 months' for follow-on formula without describing as stages (IF:1, FoF:2) | | Yes |
|  |  | Where label describes as 1 for IF and 2 for FoF as stages | | No |
| 6.3 | A warning about the health risks of improper use, preparation or storage and of introducing the product prior to the recommended age (FoF) | Clear: whether it clearly states the health risk of improper use, such as ‘Failure to follow instructions may make your baby ill’?  Conspicuous: whether it is described in different colours, fonts, or sizes.  Easily readable: whether it is easy to find and understand the texts | Where all three items were classified as Yes | Yes |
|  |  |  | - Where some of the three items were Yes, or none of them was classified as Yes  - Where it does not refer to the health risks at all | No |
| 6.4 | The required storage conditions both before and after opening, taking into account climatic conditions | Clear: whether it is explained without unambiguous  Conspicuous: whether it has a title such as 'Storage" and described in different colours, fonts, or sizes.  Easily readable: whether it is easy to find and understand the texts | Where all three items were classified as Yes | Yes |
|  |  |  | Where some of the three items were Yes, or none of them was classified as Yes | No |
| 6.5 | The name and national address of the manufacturer or distributor | Where label contains the name and national address of the manufacturer or distributor and they can be easily found | | Yes |
|  |  | Where label does not contain the information | | No |
| 7 | Does the product label avoid containing any health or nutrition claims or state or imply that a relationship exists between the product or its components and health? | Where label does not contain nutrition and health claims or imply that relationship exists between the product or its components and health | | Yes |
|  |  | Where label contains nutrition and health claims or imply that relationship exists between the product or its components and health | | No |
| 8 | Does the product label contain the following: | | | |
| 8.1 | The words, ‘IMPORTANT NOTICE’ in capital letters. and thereunder, ‘Breastfeeding is the normal and optimal way to feed infants and young children. Breastmilk is important for the healthy growth and development of infants and young children. It protects against diarrhoea and other illnesses’. | Where label contains all of four elements  - IMPORTANT NOTICE in capital letters  - Breastfeeding is the normal and optimal way  - Breastfeeding is important for healthy growth and development  - It protects against health problems such as diarrhoea and other illnesses | | Yes |
|  |  | Where label contains some of the four elements or none at all | | No |
| 8.2 | The word, ‘WARNING’ and thereunder, ‘Before deciding to supplement or replace breastfeeding with this product, seek the advice of a health professional. It is important for your baby’s health that you follow all preparation instructions carefully. If you use a feeding bottle, your baby may refuse to feed from the breast. It is more hygienic to feed from a cup’. | Where label contains all five elements  - the titile, ‘WARNING’  - Seek the advice of a health professional before using the product  - The need to follow all preparation instructions carefully  - If a bottle is used, the baby may refuse to feed from the breast  - More hygienic to feed from a cup | | Yes |
|  |  | Where label contains some of the five elements or none at all | | No |
| 8.3 | Preparation instructions:  i. Powdered formula is not sterile and may be contaminated with pathogenic microorganisms during the manufacturing process or may become contaminated during preparation  ii. It is necessary for formula to be prepared one feed at a time using water first boiled and then cooled to not less than 70 °C  iii. Any unused milk must be discarded immediately after every feed | Where label contains all three elements  - Powder formula is not sterile and may become contaminated during preparation  - Prepare one at a time and use water first boiled and then cooled  - Discard immediately after feeding | | Yes |
|  |  | Where label contains some of the three elements or none at all | | No |
|  |  | Liquid type formula milk | | NA |
| 8.4 | A feeding chart in the preparation instructions | Where label includes a feeding chart as a table | | Yes |
|  |  | Where label includes only a feeding guide as a text or none | | No |
| 9 | The product label avoid the use of the terms “maternalised”, “humanised” or similar terms or any comparison with breastmilk | Where label does not use ‘humanised’, ‘maternalised’, ‘adapted’ and their synonyms | | Yes |
|  |  | Where label does exactly use ‘humanised’, ‘maternalised’, ‘adapted’ and their synonyms | | No |
| 10 | The product label avoid the use of **text** that may tend to discourage breastfeeding | Other than those classified as No | | Yes |
|  |  | Where label contains:  - Where label contains nutrition and health claims  - Where label contains emotional text to ensure the quality of the product, to support consumers or to appeal the product or company  - Where label refers to breastfeeding or breastmilk | | No |
| 11 | The specific source of the protein | Where label refers to a specific type of protein such as ‘milk protein’, ‘whey protein from work’, ‘cow’s milk protein’, ‘casein’ | | Yes |
|  |  | Where label does not refer to a specific type of protein | | No |
| 12 | For FoF, does the product label state that it should not be used for infants less than six months old or used as the sole source of nutrition for infants? | Where follow-on formula label clearly states both:  - Not be used less than six months infant  - Not be used as the sole source of nutrition | | Yes |
|  |  | Where follow-on formula label does not include both or none | | No |

Appendix 2. Advertisement for the same line of the same brand of follow-on formula using photographs of the product on the infant formula labels

|  | Advertisement of follow-on formula on infant formula labels |
| --- | --- |
| Example 1 | 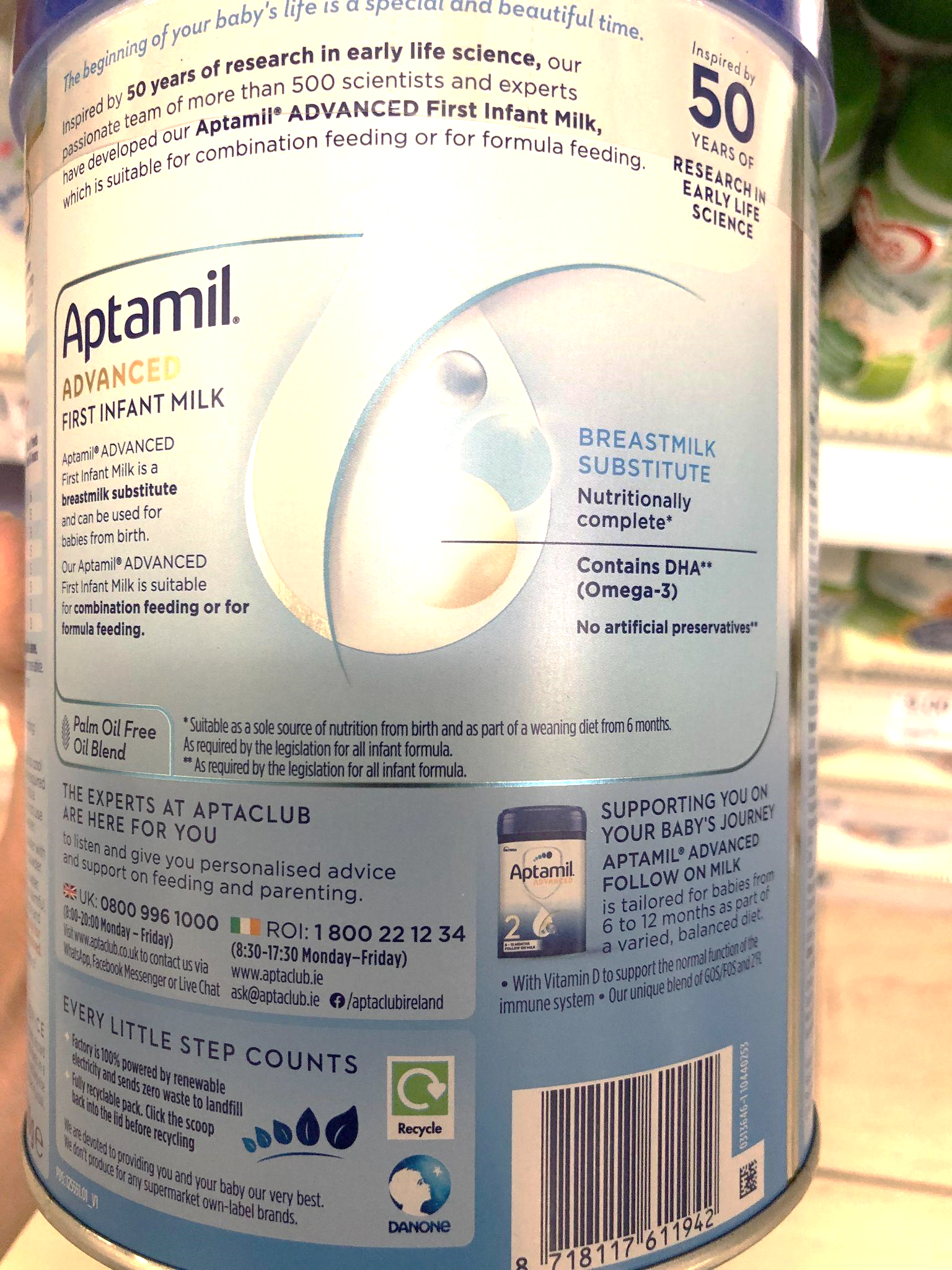 |
| Example 2 | 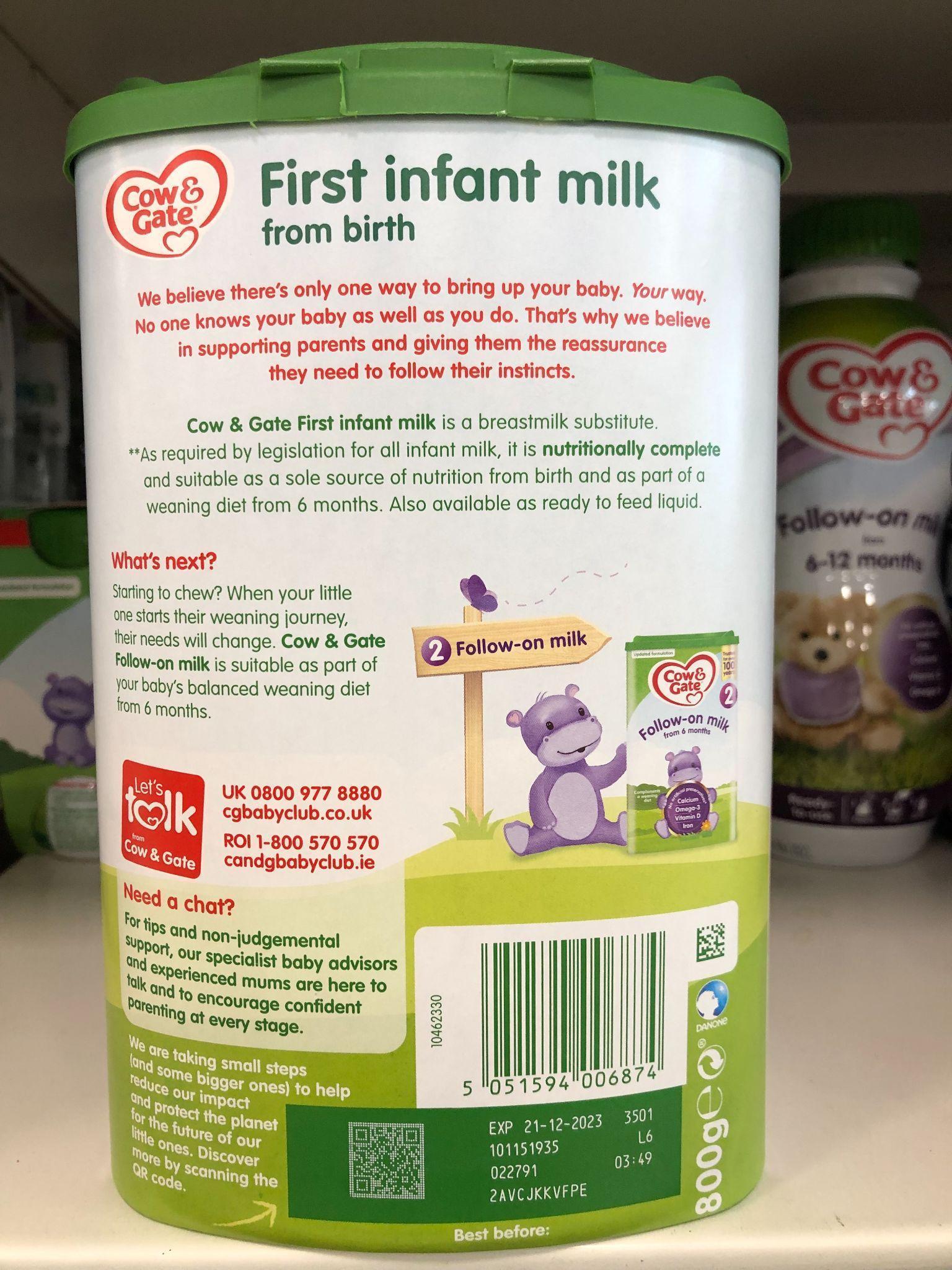 |
| Example 3 | 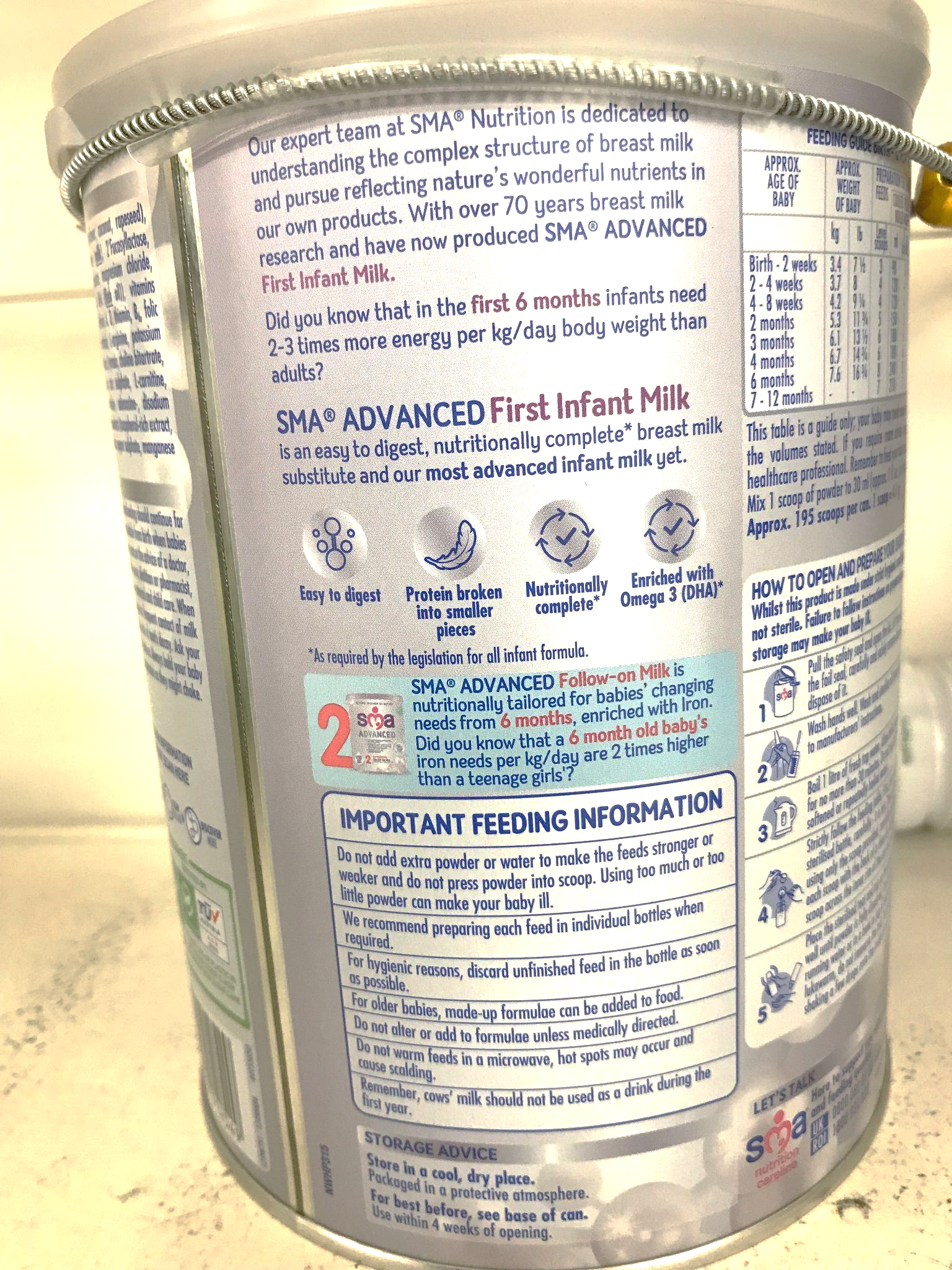 |
